# Safety and efficacy of sacubitril/valsartan vs. benazepril administered as initial treatment for STEMI patients with mid-range ejection fraction: a propensity score matching analysis

**DOI:** 10.3389/fcvm.2026.1752230

**Published:** 2026-05-28

**Authors:** Yanbo Wang, Lifang Su, Qing Zhou, Jia Tian, Wei Zhi, Yang Fu, Qing Wang, Yunfa Jiang, Xinshun Gu

**Affiliations:** The Fifth Department of Cardiology, Second Hospital of Hebei Medical University, Shijiazhuang, China

**Keywords:** heart failure with mid-range ejection fraction, left ventricular remodeling, propensity score matching, sacubitril/valsartan, ST-segment elevation myocardial infarction

## Abstract

**Objective:**

We assessed the safety and efficacy of initial treatment with sacubitril/valsartan (S/V) in STEMI patients with mid-range left ventricular ejection fraction (LVEF, 40%–49%).

**Methods:**

We consecutively enrolled STEMI patients who received successful reperfusion therapy at the Second Hospital of Hebei Medical University between January 2019 and January 2025. Clinic follow-up visits were performed at 2 weeks and 1 month after treatment initiation. PSM was used to balance baseline characteristics between the benazepril and S/V cohorts, minimizing selection bias and confounding. Echocardiographic parameters were assessed at baseline (within 24 h of symptom onset) and at the 1-month follow-up. Major adverse cardiac events (MACE) and safety outcomes were systematically reported.

**Results:**

A total of 134 eligible patients were enrolled, including 96 in the S/V cohort and 38 in the benazepril cohort. After 1:1 PSM, 35 patients were included in each matched group, with well-balanced baseline characteristics. After 1 month of treatment, the S/V group showed a significantly greater reduction in left ventricular end-systolic volume (LVESV), as well as greater improvements in left ventricular ejection fraction (LVEF) and left ventricular global longitudinal strain (GLS), compared with the benazepril group (all *P* < 0.05). There were no significant between-group differences in the incidence of major adverse cardiac events (MACE) or safety endpoints (all *P* > 0.05).

**Conclusion:**

In STEMI patients with mid-range LVEF, initial S/V and benazepril treatment had comparable safety profiles. S/V showed superior efficacy in improving short-term cardiac systolic function, as measured by surrogate echocardiographic endpoints. These preliminary findings require validation in larger, prospective randomized controlled trials with longer follow-up to confirm whether these benefits translate into improved long-term clinical outcomes.

## Introduction

ST-segment elevation myocardial infarction (STEMI), a life-threatening acute coronary syndrome, is characterized by acute cardiomyocyte necrosis caused by prolonged ischemia secondary to complete coronary artery occlusion (1). Despite timely and successful reperfusion therapy, up to 25% of STEMI patients develop subsequent heart failure (HF), which is strongly associated with increased mortality and poor long-term prognosis (2, 3).

HF with mid-range ejection fraction (HFmrEF), defined as left ventricular ejection fraction (LVEF) of 40%–49%, is categorized as an intermediate phenotype between HF with reduced EF (HFrEF, LVEF < 40%) and HF with preserved EF (HFpEF, LVEF ≥ 50%). Historically, HFmrEF has been grouped with HFpEF in most clinical trials, while empirically treated with guideline-directed medical therapy (GDMT) established for HFrEF (4). Due to the limited dedicated research on HFmrEF, the optimal pharmacological strategy for STEMI patients with mid-range LVEF remains poorly defined, representing a critical unmet clinical need.

Sacubitril/valsartan (S/V), a first-in-class angiotensin receptor-neprilysin inhibitor (ARNI), simultaneously inhibits the renin-angiotensin-aldosterone system (RAAS) and enhances the natriuretic peptide system. The landmark PARADIGM-HF trial demonstrated that S/V significantly reduced cardiovascular death and HF hospitalization compared with enalapril in patients with chronic HFrEF (5, 6). Subsequent studies have shown that S/V more effectively attenuates adverse ventricular remodeling compared with conventional angiotensin-converting enzyme inhibitors (ACEIs) in patients after acute myocardial infarction (AMI) (7). However, the PARADISE-MI trial, which compared S/V with ramipril in high-risk post-AMI patients with LVEF ≤40%, failed to show the superiority of S/V for the primary composite endpoint of cardiovascular death or HF events. Furthermore, a pre-specified analysis by Docherty et al. (Circulation 2021) found no significant difference in LV remodeling between S/V and valsartan in post-AMI patients with LV systolic dysfunction.

Notably, most prior studies focused on patients with HFrEF, and data on the early initiation of S/V in STEMI patients with HFmrEF remain scarce. Therefore, this retrospective, PSM analysis aimed to evaluate the safety and efficacy of initial S/V versus benazepril treatment in STEMI patients with mid-range LVEF, to provide preliminary evidence for early pharmacological optimization in this understudied population.

## Methods

### Study population

We consecutively enrolled patients with STEMI who received successful reperfusion therapy at the Second Hospital of Hebei Medical University between January 16, 2019 and January 27, 2025.

Inclusion criteria: (1) age >18 years; (2) first STEMI event, diagnosed according to universal myocardial infarction definition guidelines (8), with symptom onset within 12 h; (3) successful reperfusion therapy, including primary percutaneous coronary intervention (PCI), successful PCI within 12–24 h after successful thrombolysis, or successful rescue PCI; (4) no contraindications to S/V or benazepril; (5) LVEF of 40%–49% at baseline (within 24 h of symptom onset); (6) high-quality echocardiographic images obtained at baseline and 1-month follow-up.

Exclusion criteria: (1) prior myocardial infarction; (2) significant hypertrophic cardiomyopathy, valvular heart disease, or dilated cardiomyopathy; (3) active infection, malignancy, or inflammatory disease (e.g., active rheumatic disease); (4) hospital presentation >12 h after symptom onset; (5) severe pulmonary hypertension; (6) cardiogenic shock; (7) thrombolysis in myocardial infarction (TIMI) flow grade 0–1 after primary PCI.

The study protocol was approved by the Ethics Committee of the Second Hospital of Hebei Medical University (2020-R672-R01-R701-R01) and conducted in accordance with the Declaration of Helsinki. Written informed consent was waived by the ethics committee due to the retrospective, non-interventional design of the study.

### Treatment protocol

Study drugs were initiated within 24 h of symptom onset after successful reperfusion. S/V was started at an oral dose of 25 mg twice daily, with uptitration to 50–100 mg twice daily at the 2-week follow-up if tolerated. Benazepril was initiated at 5 mg once daily, with uptitration to 10 mg once daily at 2 weeks if tolerated. Treatment allocation was determined by the attending cardiologist based on individualized patient factors, including blood pressure, renal function, and expected tolerability, with no mandatory institutional protocol for drug selection.

Concomitant GDMT, including beta-blockers, sodium-glucose cotransporter 2 inhibitors (SGLT2i), mineralocorticoid receptor antagonists (MRAs), and statins, was initiated within 24 h when clinically indicated, with uptitration to the maximum tolerated dose during follow-up. Dosing and uptitration of all concomitant GDMT were standardized per current STEMI and HF guidelines, with no between-group differences in target dosing protocols. Medication adherence was assessed via self-report and pill count at each follow-up visit, with no significant difference in adherence rates between the two groups (both >95% at 1 month).

### Angiographic and laboratory assessments

Coronary angiographic findings, including infarct-related artery (IRA) distribution and thrombus burden, were documented. Stent implantation, thrombus aspiration, and GPIIb/IIIa receptor antagonist use were determined by the interventional cardiologist during the procedure. Successful reperfusion was defined as TIMI flow grade 3 with residual stenosis <30% in the IRA (9).

High-sensitivity troponin I, creatine kinase, and creatine kinase-MB levels were measured at admission, 6 h after primary PCI, and daily for up to 3 days. N-terminal pro-B-type natriuretic peptide (NT-proBNP) was measured at baseline (within 24 h of symptom onset) and at the 1-month follow-up visit.

### Echocardiographic assessment

Complete transthoracic echocardiography was performed within 24 h of symptom onset and repeated at the 1-month follow-up, using a Philips Epiq 7c ultrasound system with an S5-1 transducer (Philips Medical Systems, Andover, MA, USA) (10). All examinations were performed by an experienced cardiologist blinded to treatment allocation.

LVEF was measured using the biplane Simpson's method. Doppler parameters, including peak early diastolic mitral inflow velocity (E), peak late diastolic mitral inflow velocity (A), and mean early diastolic mitral annular velocity (e'), were obtained from the apical 4-chamber view with the sample volume positioned at the mitral leaflet tips. LV global longitudinal strain (GLS) was analyzed via 2D speckle-tracking echocardiography from apical 2-, 3-, and 4-chamber views, with end-systole timed to aortic valve closure. Endocardial borders were manually traced at end-systole for strain analysis (11, 12).

Reproducibility of GLS measurements was assessed via intra-observer and inter-observer variability. 10% of randomly selected studies were re-analyzed by the same observer (intra-observer) and a second independent blinded observer (inter-observer) at least 4 weeks apart. Variability was quantified using intraclass correlation coefficients (ICCs), with excellent reproducibility defined as ICC >0.80.

### Follow-up and outcome definitions

All patients were followed up at the outpatient clinic at 2 weeks and 1 month after treatment initiation, by clinicians blinded to the study treatment allocation. Laboratory assessments were performed at both visits.

The primary efficacy outcomes were changes in echocardiographic parameters of cardiac systolic and diastolic function from baseline to 1 month, and the incidence of MACE during follow-up. MACE was defined as a composite of target vessel revascularization, life-threatening ventricular arrhythmia, recurrent angina requiring hospitalization, new-onset or worsening HF, and all-cause mortality. This composite endpoint was selected to capture both ischemic and HF-related events relevant to the early post-STEMI period, consistent with prior studies of early pharmacotherapy in this population.

Safety outcomes included symptomatic hypotension, hyperkalemia (serum potassium ≥5.5 mmol/L), worsening renal function (increase in serum creatinine ≥0.5 mg/dL [≥44 µmol/L] and/or reduction in estimated glomerular filtration rate ≥25%), and angioedema. Symptomatic hypotension was defined as systolic blood pressure ≤90 mmHg and/or diastolic blood pressure ≤60 mmHg accompanied by clinical signs of hypoperfusion.

### Propensity score matching

PSM was performed to minimize selection bias and confounding between the S/V and benazepril cohorts. A logistic regression model was used to generate propensity scores, with the following pre-specified covariates included in the model: age, sex, hypertension, diabetes mellitus, current smoking, alcohol consumption, reperfusion strategy, and concomitant in-hospital medications (beta-blockers, SGLT2i, MRAs, statins).

One-to-one matching without replacement was performed using a greedy matching algorithm, with a caliper width of 0.05 of the standard deviation of the logit of the propensity score. Covariate balance before and after matching was assessed using both standardized mean differences (SMD) and Student's *t*-tests/Wilcoxon rank-sum tests for continuous variables, and chi-squared tests/Fisher's exact tests for categorical variables. SMD <0.1 was considered to indicate excellent balance between groups.

### Statistical analysis

Continuous variables are presented as mean ± standard deviation (SD) for normally distributed data, or median (interquartile range, IQR) for non-normally distributed data. Categorical variables are presented as counts (percentages).

For the matched cohort, paired statistical tests were used to account for the paired nature of the data: paired t-tests for normally distributed continuous variables, Wilcoxon signed-rank tests for non-normally distributed continuous variables, and McNemar's test for categorical variables. For the pre-matched cohort, independent samples *t*-tests, Mann–Whitney U tests, chi-squared tests, or Fisher's exact tests were used as appropriate.

A *post-hoc* power analysis was performed to assess the statistical power of the primary echocardiographic endpoint (change in LVEF) using the observed effect size from the matched cohort. A two-sided *P*-value <0.05 was considered statistically significant. All statistical analyses were performed using R version 4.3.1 (R Foundation for Statistical Computing, Vienna, Austria).

## Results

### Baseline characteristics before and after PSM

A total of 134 eligible patients were enrolled, including 96 patients in the S/V group and 38 patients in the benazepril group. After 1:1 PSM, 35 patients were included in each matched group.

Baseline characteristics of the pre- and post-matched cohorts are shown in Table 1. Before matching, there were no significant differences in baseline demographics, medical history, peak myocardial injury markers, or concomitant medication use between the two groups (all *P* > 0.05). After matching, all baseline characteristics remained well-balanced, with all SMD values <0.1 and no significant between-group differences (all *P* > 0.05), confirming adequate covariate balance.

**Table 1:** Baseline characteristics between the two groups before and after PSM.

Characteristic	Before PSM				After PSM			
	ACEI group (*n* = 38)	ARNI group (*n* = 96)	*P* value	SMD	ACEI group (*n* = 35)	ARNI group (*n* = 35)	*P* value	SMD
Age (year)	60.58 ± 12.30	57.19 ± 14.03	0.194	0.255	60.23 ± 12.33	59.82 ± 12.32	0.885	0.033
Male - no (%)	30 (78.9)	80 (83.3)	0.551	0.115	27 (77.1)	28 (80.0)	0.771	0.069
BMI (Kg/m^2^)	25.94 ± 4.35	26.33 ± 3.76	0.608	0.096	26.1 ± 4.47	25.73 ± 3.60	0.703	0.091
Anterior wall - no (%)	23 (60.5)	72 (75.0)	0.096	0.319	22 (62.9)	24 (68.6)	0.615	0.120
Killip ≥ II - no (%)	15 (39.5)	29 (30.2)	0.303	0.198	12 (34.3)	10 (28.6)	0.607	0.124
Heart rate (bpm)	77.95 ± 13.71	81.96 ± 17.57	0.209	0.251	79.06 ± 13.37	80.77 ± 19.25	0.667	0.101
SBP (mmHg)	126.95 ± 23.20	127.51 ± 18.01	0.881	0.027	128.71 ± 23.17	125.23 ± 20.32	0.506	0.159
DBP (mmHg)	82.16 ± 15.19	84.45 ± 14.19	0.411	0.155	83.69 ± 14.81	83.40 ± 13.51	0.933	0.020
Onset-FMC (min)	120 (60.0, 337.5)	105 (60,180)	0.286	0.172	120 (60,360)	150 (60,300)	0.698	0.065
Total ischemia time (min)	480 (180,720)	300 (127.5, 565.0)	0.274	0.180	480 (180,660)	360 (180,680)	0.579	0.087
cTnI peak (ng/L)	100000 (100000,125000)	100000 (100000,125000)	0.373	0.102	101000 (100000,125000)	100000 (95197,121350)	0.341	0.122
CK peak (U/L)	3814.50 (2361.00,4790.75)	3593.00 (1865.00,5739.75)	0.645	0.058	3797 (2301,4464)	3755 (1895,3568)	0.919	0.015
CK-MB peak (U/L)	372.50 (233.75,510.00)	320.50 (208.75,569.50)	0.850	0.023	367 (230,506)	366 (199,601)	0.680	0.059
Hypertension - no (%)	20 (52.6)	46 (47.3)	0.703	0.107	18 (51.4)	16 (45.7)	0.632	0.114
T2DM - no (%)	4 (10.5)	23 (24.0)	0.097	0.349	4 (11.4)	8 (22.9)	0.342	0.297
Hyperlipidemia - no (%)	2 (5.3)	6 (6.3)	1.000	0.044	2 (5.7)	0 (0)	0.493	0.247
CKD - no (%)	0 (0)	3 (3.1)	0.558	0.180	0 (0)	2 (5.7)	0.493	0.247
CHD family history - no (%)	4 (10.5)	8 (8.3)	0.740	0.079	4 (11.4)	2 (5.7)	0.673	0.194
Smoke - no (%)	15 (39.5)	37 (38.5)	0.921	0.020	15 (42.9)	13 (37.1)	0.626	0.119
IRA - no (%)			0.553				0.217	
LM	0 (0)	1 (1.0)			0 (0)	1 (2.9)		
LAD	24 (63.2)	70 (72.9)			23 (65.7)	22 (62.9)		
LCX	3 (7.9)	5 (5.2)			3 (8.6)	0 (0)		
RCA	11 (28.9)	20 (20.8)			9 (25.7)	12 (34.3)		
Multivessel - no (%)	24 (63.2)	58 (60.4)	0.769	0.058	21 (60.0)	23 (65.7)	0.621	0.118
PCI - no (%)	32 (84.2)	78 (81.3)	0.687	0.078	30 (85.7)	29 (82.9)	0.743	0.077
Stent	1 (0,1)	1 (0,1)	0.910	0.014	1 (0,1)	1 (0,1)	0.648	0.064
Complete revascularization - no (%)	18 (47.4)	40 (41.7)	0.548	0.116	18 (51.4)	13 (37.1)	0.229	0.289
Aspirin - no (%)	35 (92.1)	87 (87.4)	1.000	0.159	32 (91.4)	32 (91.4)	1.000	0.000
P2Y12 receptor inhibitor - no (%)	36 (94.7)	90 (93.8)	1.000	0.037	33 (94.3)	35 (100.0)	0.493	0.247
Statin - no (%)	34 (89.4)	91 (94.8)	0.145	0.209	31 (88.6)	33 (94.3)	0.405	0.200
*β*-blocker - no (%)	30 (78.9)	92 (95.8)	0.004	0.571	29 (82.9)	34 (97.1)	0.106	0.510
Spinolactone - no (%)	22 (57.9)	69 (71.9)	0.118	0.301	20 (57.1)	25 (71.4)	0.212	0.305
SGLT2i - no (%)	14 (36.8)	55 (57.3)	0.033	0.417	14 (40.0)	16 (45.7)	0.629	0.116
Vasodilator - no (%)	20 (52.6)	67 (69.8)	0.061	0.361	18 (51.4)	24 (68.6)	0.143	0.356
Diuretic - no (%)	27 (71.1)	83 (86.5)	0.036	0.414	24 (68.6)	30 (85.7)	0.088	0.423

BMI, body mass index; SBP, systolic blood pressure; DBP, diastolic blood pressure; FMC, first medical contact; cTnI, cardiac troponin I; CK, creatine kinase; CK-MB, creatine kinase-MB; T2DM, type 2 diabetes mellitus; CKD, chronic kidney disease; CHD, coronary heart disease; IRA, infarct-related artery; LM, left main; LAD, left anterior descending; LCX, left circumflex; RCA, right coronary artery; PCI, percutaneous coronary intervention; SGLT2i, sodium-glucose cotransporter 2 inhibitor; SMD, standardized mean difference. SMD <0.1 indicates excellent covariate balance between groups.

### Echocardiographic parameters at baseline and 1-month follow-up

Echocardiographic parameters for the matched cohort are presented in Table 2. At baseline (within 24 h of symptom onset), there were no significant between-group differences in any systolic or diastolic function parameters (all *P* > 0.05).

**Table 2:** Comparison of echocardiographic results between the two groups.

Parameter	Within 24 h of symptom onset			After 1 month of treatment		
	ACEI group (*n* = 35)	ARNI group (*n* = 35)	*P* value	ACEI group (*n* = 35)	ARNI group (*n* = 35)	*P* value
LAD (mm)	33.80 ± 3.78	33.63 ± 5.57	0.881	35.92 ± 3.16	35.06 ± 3.79	0.785
LVEDV (mL)	115.90 ± 25.47	117.55 ± 26.00	0.789	122.11 ± 8.25	122.91 ± 13.58	0.839
LVESV (mL)	62.36 ± 13.89	62.71 ± 14.84	0.918	62.86 ± 8.81	57.57 ± 8.13	0.011
SV (mL)	51.39 ± 15.20	57.38 ± 12.92	0.098	59.26 ± 10.57	65.34 ± 13.03	0.045
LVEF (%)	46.66 ± 4.11	45.05 ± 2.96	0.065	47.85 ± 8.25	52.89 ± 6.40	0.006
E/e'	11.25 ± 3.30	11.34 ± 3.63	0.914	11.02 ± 3.59	11.31 ± 4.09	0.753
E/A	0.94 ± 0.37	0.81 ± 0.34	0.142	0.99 ± 0.37	0.93 ± 0.29	0.450
Diastolic function classification - no (%)			0.844			0.587
0	3 (8.6)	2 (5.7)		6 (17.1)	4 (11.4)	
1	22 (62.9)	24 (68.6)		21 (60.0)	21 (60.0)	
2	10 (28.6)	9 (25.7)		7 (20.0)	10 (28.6)	
3	0 (0)	0 (0)		1 (2.9)	0 (0)	
Global longitudinal strain (%)	−11.26 ± 2.42	−11.54 ± 2.56	0.633	−10.51 ± 2.78	−13.80 ± 2.72	<0.001

LAD, left atrial diameter; LVEDV, left ventricular end-diastolic volume; LVESV, left ventricular end-systolic volume; SV, stroke volume; LVEF, left ventricular ejection fraction; E, peak velocity of the early diastolic wave; e', average early diastolic mitral annular velocity; A, peak flow velocity of the atrial systolic wave.

At the 1-month follow-up, the S/V group had a significantly lower LV end-systolic volume (57.57 ± 8.13 mL vs. 62.86 ± 8.81 mL, *P* = 0.011) compared with the benazepril group. The S/V group also showed significantly greater improvements in stroke volume (SV) and LVEF compared with the benazepril group (all *P* < 0.05). Both groups had significant improvements in SV and LVEF from baseline to 1 month.

There were no significant between-group differences in diastolic function parameters (E/e', E/A ratio) at baseline or follow-up (all *P* > 0.05). However, the S/V group had a significantly greater improvement in LV GLS compared with the benazepril group (−13.80% ± 2.72% vs. −10.51% ± 2.78%, *P* < 0.001). Intra-observer and inter-observer ICCs for GLS measurements were 0.92 (95% CI 0.85−0.96) and 0.89 (95% CI 0.81−0.94), respectively, indicating excellent reproducibility.

### Changes in NT-proBNP levels

NT-proBNP levels were well-balanced between the two groups at baseline (*P* > 0.05). After 1 month of treatment, NT-proBNP levels were significantly reduced in both groups compared with baseline (all *P* < 0.001), with the S/V group showing significantly lower 1-month NT-proBNP levels than the benazepril group (between-group *P* < 0.001) (Table 3).

**Table 3:** Comparison of level of NT-proBNP between the two groups.

Time point	ACEI group (*n* = 35)	ARNI group (*n* = 35)	*P* value
NT-proBNP at baseline (pg/mL)	1538.26 ± 674.00	1348.49 ± 531.43	0.196
NT-proBNP at 1-month follow-up (pg/mL)	796.8 ± 156.1^a^	492.7 ± 188.8^a^	<0.001

a *P* < 0.001 for within-group comparison between baseline and 1-month follow-up.

### MACE outcomes during follow-up

The incidence of MACE during the 1-month follow-up is shown in Table 4. There were 8 MACE events in the S/V group and 9 MACE events in the benazepril group, with no significant between-group difference in the overall incidence of MACE (22.9% vs. 25.7%, *P* = 0.791, McNemar's test). There were also no significant differences in the individual components of MACE between the two groups (all *P* > 0.05).

**Table 4:** Comparison of MACE between two groups during follow-up.

Event	ACEI group (*n* = 35)	ARNI group (*n* = 35)	*P* value
MACE - no (%)	9 (25.7)	8 (22.9)	0.791
Cardiogenic shock - no (%)	1 (2.9)	1 (2.9)	1.000
Malignant arrhythmia	2 (5.7)	4 (11.4)	0.673
Target vessel revascularization - no (%)	0 (0)	0 (0)	–
All-cause death - no (%)	1 (2.9)	0 (0)	1.000
Recurrent angina requiring hospitalization - no (%)	5 (14.3)	3 (8.6)	0.710

MACE: major adverse cardiac events.

### Safety outcomes

Safety endpoint events during follow-up are summarized in Table 5. There were 5 safety events in the S/V group and 7 safety events in the benazepril group, with no significant between-group difference in the overall incidence of safety endpoints (14.3% vs. 20.0%, *P* = 0.546, McNemar's test). No cases of angioedema occurred in either group.

**Table 5:** Comparison of safety endpoint events between two groups during follow-up.

Adverse event	ACEI group (*n* = 35)	ARNI group (*n* = 35)	*P* value
Adverse effects - no (%)	7 (20.0)	5 (14.3)	0.546
Symptomatic hypotension	1 (2.9)	2 (5.7)	1.000
Angioedema - no (%)	0 (0)	0 (0)	–
Cough - no (%)	4 (11.4)	0 (0)	0.114
Hyperkalemia - no (%)	0 (0)	0 (0)	–
Worsening renal function - no (%)	2 (5.7)	3 (8.6)	1.000

### Power analysis

*Post-hoc* power analysis for the primary endpoint of change in LVEF showed that the matched cohort (35 patients per group) achieved 92% statistical power to detect the observed between-group difference in LVEF change (3.33% absolute difference) at a two-sided alpha level of 0.05. For MACE and safety endpoints, the study had limited power to detect small between-group differences, with a 32% power to detect a 50% relative reduction in MACE incidence, indicating a high risk of type II error for these clinical endpoints. This means that we cannot rule out a clinically meaningful difference in MACE with larger sample sizes and longer follow-up.

## Discussion

In this retrospective PSM analysis of STEMI patients with mid-range LVEF, we found that initial treatment with S/V significantly improved short-term echocardiographic parameters of cardiac systolic function, including LVESV, LVEF, and LV GLS, compared with benazepril. The two treatments had comparable safety profiles, with no significant differences in the incidence of MACE or adverse events during the 1-month follow-up. These preliminary findings provide important early evidence for the use of S/V in the understudied HFmrEF population post-STEMI.

Adverse LV remodeling, driven by cardiomyocyte necrosis, neurohormonal activation, and RAAS overstimulation, is the key pathophysiological mechanism underlying HF development after STEMI (13). Even after successful reperfusion and TIMI 3 flow restoration, early myocardial stunning and LV systolic dysfunction occur within 24–72 h of STEMI onset, making early neurohormonal inhibition critical to prevent progressive remodeling (14–16). ACEIs are a cornerstone of GDMT for post-STEMI patients, with benazepril shown to attenuate ventricular remodeling in preclinical and clinical studies of AMI. S/V, via dual RAAS inhibition and natriuretic peptide enhancement, has more potent anti-remodeling effects than ACEIs in HFrEF populations (17–19). However, its efficacy in STEMI patients with HFmrEF remains poorly defined.

Our findings build on and contextualize prior landmark trials of S/V in post-AMI patients. The PARADISE-MI trial compared S/V with ramipril in 5661 high-risk post-AMI patients with LVEF ≤40% and pulmonary congestion, and found no significant reduction in the primary composite endpoint of cardiovascular death or HF events. In contrast, our study focused on patients with mid-range LVEF (40%–49%), a population excluded from PARADISE-MI, and found significant benefits of S/V on surrogate markers of ventricular remodeling (20). This discrepancy suggests that the benefits of S/V may differ across the LVEF spectrum, and that the treatment effect observed in our study is a drug-specific effect in HFmrEF, rather than a class effect of RAAS inhibition.

Similarly, Docherty et al. reported no significant difference in LV remodeling between S/V and valsartan in post-AMI patients with LV systolic dysfunction in a pre-specified analysis of the PARADISE-MI trial (21). Several factors may explain the differences between their findings and ours. First, their study included patients with LVEF ≤40%, while our cohort had mid-range LVEF, where the potential for reverse remodeling may differ. Second, their follow-up duration was longer (6 months), while our study focused on early changes at 1 month, which may capture the acute phase of post-infarct remodeling. Third, our comparator was benazepril (an ACEI), while theirs was valsartan (an ARB), with different mechanisms of RAAS inhibition.

Our study also found that S/V significantly reduced NT-proBNP levels to a greater extent than benazepril. NT-proBNP is a well-established biomarker of LV wall stress and volume overload, and a strong predictor of outcomes in HF patients. The greater reduction in NT-proBNP with S/V suggests that it more effectively reduces LV filling pressures and volume overload, thereby alleviating HF pathophysiology in patients with HFmrEF (14–16).

LV GLS, a more sensitive marker of myocardial contractility than LVEF, has emerged as a powerful predictor of reverse remodeling and clinical outcomes in post-AMI patients (12, 22). Our study found that S/V significantly improved LV GLS compared with benazepril, with excellent reproducibility of strain measurements. This finding indicates that S/V provides more comprehensive benefits on myocardial function beyond LVEF, which may translate into sustained benefits on ventricular remodeling with longer follow-up.

Notably, the improvements in LVESV, LVEF, and LV GLS observed in this study are well-established surrogate markers of adverse ventricular remodeling and long-term clinical outcomes in post-STEMI patients. Prior studies have consistently demonstrated that even modest improvements in LVEF and absolute reductions in LVESV within the first 1-3 months post-STEMI are associated with significant reductions in 5-year cardiovascular mortality and HF hospitalization risk. Furthermore, worse LV GLS post-STEMI is an independent predictor of major adverse cardiac events, even in patients with preserved or mid-range LVEF (12, 22). While our study is limited to short-term follow-up, these early improvements in validated surrogate endpoints provide a strong biological rationale for potential long-term clinical benefits of early S/V initiation in this population.

Notably, we found no significant between-group differences in MACE or safety outcomes. This is likely due to the small sample size and very short 1-month follow-up duration, which limited the statistical power to detect differences in clinical events. *Post-hoc* power analysis confirmed a high risk of type II error for these endpoints, meaning that we cannot rule out a clinically meaningful difference in MACE with larger sample sizes and longer follow-up. Importantly, the safety profile of S/V was comparable to benazepril, with no increased risk of hypotension, hyperkalemia, or renal dysfunction, supporting the tolerability of early S/V initiation in this acute STEMI population.

Recent meta-analyses have also shown that S/V reduces ventricular arrhythmic burden in patients with HFrEF, including a systematic review and meta-analysis by Pozzi et al. which demonstrated that S/V significantly reduced the incidence of malignant ventricular arrhythmias, appropriate implantable cardioverter-defibrillator shocks, and sudden cardiac death in HFrEF populations (23). This anti-arrhythmic effect may be mediated by improved ventricular remodeling, reduced myocardial wall stress, and attenuation of neurohormonal activation, all of which are highly relevant to the post-STEMI population, who are at elevated risk of life-threatening arrhythmias in the early infarct period. While our study had too few arrhythmic events to formally assess this endpoint in our HFmrEF cohort, this potential additional benefit of S/V warrants further investigation in larger, prospective studies.

### Limitations

Our study has several important limitations that must be acknowledged. First, it was a single-center, retrospective observational study, which introduces inherent selection bias. Although PSM was used to balance baseline covariates, residual confounding may persist due to unmeasured factors that were not included in the propensity score model. These factors include, but are not limited to, differences in patient medication adherence beyond the 1-month follow-up, subtle variations in post-discharge cardiac rehabilitation and secondary prevention strategies, unreported socioeconomic factors that may influence care access and outcomes, and individual physician preferences for uptitration of GDMT. While we observed no significant differences in in-hospital and 1-month medication use between the matched groups, these unmeasured factors may still influence the observed treatment effects, and the potential for confounding by indication cannot be fully eliminated in this observational retrospective design.

Second, the sample size after PSM was small (35 patients per group), which limited the statistical power for clinical endpoints such as MACE and safety outcomes. We have provided a *post-hoc* power analysis and discussed the risk of type II error when interpreting the non-significant findings for these endpoints. Additionally, while we included all concomitant GDMT in the PSM model and confirmed no significant between-group differences in medication use after matching, we did not systematically collect data on the maximum tolerated dose of each concomitant medication for all patients, which may represent an additional source of unmeasured confounding.

Third, the follow-up duration was only 1 month, which is too short to assess the sustained effects of S/V on ventricular remodeling or long-term clinical outcomes. We have moderated our conclusions to emphasize that the findings are preliminary and limited to short-term surrogate endpoints, and that longer follow-up is required to confirm clinical benefits.

Fourth, the study was not a randomized controlled trial (RCT), which limits the causal inference that can be drawn from the findings. Our results should be considered hypothesis-generating, and require validation in large, prospective, multicenter RCTs.

## Conclusion

In STEMI patients with mid-range LVEF (40%–49%), initial treatment with S/V was associated with significantly greater improvements in short-term echocardiographic markers of cardiac systolic function and reverse remodeling, compared with benazepril. The two treatments had comparable safety and tolerability profiles. These preliminary findings support the early use of S/V in this understudied population, but require validation in larger, randomized controlled trials with longer follow-up to confirm whether these benefits on surrogate endpoints translate into improved long-term clinical outcomes.

## Data Availability

The raw data supporting the conclusions of this article will be made available by the authors, without undue reservation.
